# Photochromic derivatives of indigo: historical overview of development, challenges and applications

**DOI:** 10.3762/bjoc.20.23

**Published:** 2024-02-07

**Authors:** Gökhan Kaplan, Zeynel Seferoğlu, Daria V Berdnikova

**Affiliations:** 1 Department of Chemistry, Faculty of Science, Gazi University, Yenimahalle, Ankara, 06560, Turkey; 2 Sanko Tekstil İşletmeleri, Sanayi ve Ticaret A.Ş. Isko Sb, Bursa, 16400, Bursa, Turkey; 3 Organische Chemie II, Universität Siegen, Adolf-Reichwein-Str. 2, 57076 Siegen, Germany

**Keywords:** indigoid dyes, photochemistry, photophysics, photoswitching, *E*–*Z* isomerization

## Abstract

The importance of indigo dyes is constantly increasing with the evolution of novel textile materials and photochromic material technologies. The aim of this review article is to provide a comprehensive overview of the development of photochromic indigo derivatives from the first report on the photochromic *N,N'*-diacetylindigo in 1954 until now. We begin with the list of historical milestones in the development of photochromic indigo derivatives. Further, we provide a brief description of the synthetic procedures utilised to obtain indigo and its derivatives, outline the structural peculiarities, photophysical and photochemical properties of indigo and proceed with the detailed discussion of the photochromic indigo derivatives. Finally, we highlight the photochromism of the structural isomers of indigo (isoindigo and indirubin) and provide an overview of prospective applications of indigo photoswitches.

## Review

### Historical milestones in the development of photochromic indigo derivatives

- 1954: first reported photochromic indigo derivative – *N,N'*-diacetylindigo
- 1956: first report on the photochromism of *N,N'*-dimethylindigo
- 1956–1978: extension of the range of photochromic *N,N'*-dialkyl and *N,N'*-diacetylindigos and development of experimental and theoretical approaches towards the characterisation of the photoisomerization of indigo
- 1979–1984: development of photochromic *N,N'*-diacyl derivatives of indigo, first examples of photochromic indigos containing aromatic *N,N'*-substituents
- 1984: first report on intra- and intermolecularly bridged photochromic *N,N'*-substituted indigos (follow-up studies in 1989, 2021)
- 1985: detailed kinetic studies of the thermal backward *Z–E* isomerization of *N,N'*-substituted indigos
- Since 1980s: detailed mechanistic and structural studies of the photoisomerization of indigo
- 2015: first report on photochromism of *N,N'*-diBOC indigos (follow-up studies in 2019, 2021)
- 2017: rationalisation of the design of indigo photochromes: symmetrical and unsymmetrical *N,N'*-substituted alkyl- and arylindigos with tunable thermal half-lives (follow-up mechanistic studies in 2022)
- 2018: first report on photochromic monoarylated indigos

### Synthesis of indigo derivatives

**Figure 1:**
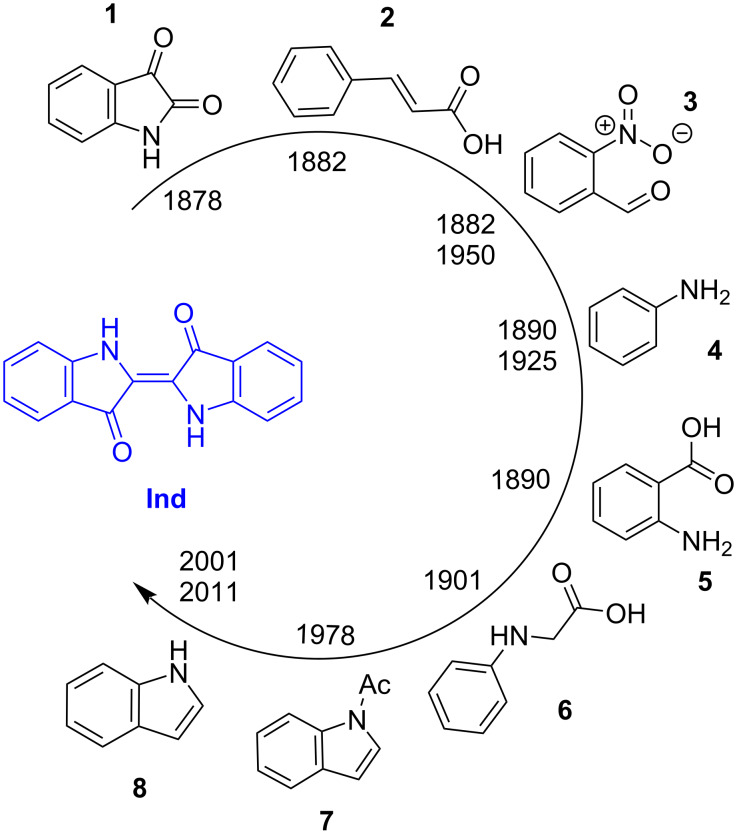
Precursors used in the synthesis of indigo [4].

Indigo (**Ind**) is one of the oldest organic molecules, which has been used as a dye for 6000 years [1]. Initially, indigo has been extracted from the plant species, for example *Indigofera tinctoria* and *Polygonum tinctorium* in Asia and America, *Isatis tinctoria* or dyer’s woad in Europe [2] before the chemical synthesis of indigo was achieved by the German chemist Adolf von Baeyer [3]. After the determination of the molecular structure of indigo in 1883, various precursors such as isatin (**1**), cinnamic acid (**2**), 2-nitrobenzaldehyde (**3**), aniline (**4**), 2-aminobenzoic acid (**5**), phenylglycine (**6**), 1-(1*H*-indol-1-yl)ethan-1-one (**7**) and indole (**8**) have been used in the synthesis (Figure 1) [4]. As an alternative to the chemical synthesis of indigo, environmentally friendly biological approaches towards constructing the indigo scaffold are also being developed for nearly a century [5–8]. The biosynthetic methods include production of indigo by microorganisms and enzymatic synthesis using heme-containing oxygenases (cytochrome P450 monooxygenases, styrene/indole monooxygenases, flavin-containing monooxygenases, Baeyer–Villiger monooxygenases, etc.) or non-heme iron oxygenases (naphthalene dioxygenases, multicomponent phenol hydroxylases) [5–8]. The synthetic approaches towards indigo and its derivatives have been recently reviewed in detail by Hecht and Huang [9].

### Strucutre and photophysical properties of indigo

**Figure 2:**
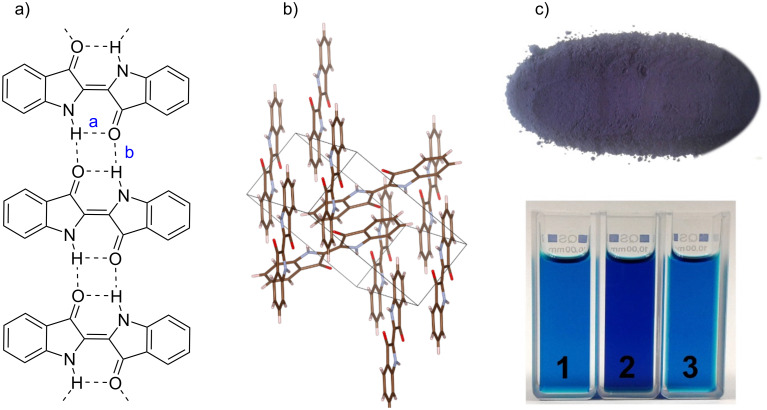
a) Intramolecular (a = 2.26 Å) and intermolecular (b = 2.11 Å) hydrogen bonds in indigo, b) crystal packing of indigo in the solid state obtained from the single-crystal X-ray diffraction data, CCDC 796873 [12], c) photos of indigo in the solid state and solutions of indigo in 1) DMSO, 2) DMF, 3) *N*-methyl-2-pyrrolidone.

Indigo dye is blue crystalline powder, which starts to melt at above 390 °C and sublimes in vacuum at above 170 °C [2]. In 1980, it was discovered that indigo exists in two crystalline modifications, namely indigo A and indigo B. In particular, 10% of indigo B was found alongside the known form indigo A (90%), in the dye crystals grown from vapor at 10 torr. Both crystalline forms of indigo have the same symmetry and similar cell parameters with the only significant difference in the values of β-angles, which results in some differences in the densities [10]. The solubility of crystalline indigo is poor even in polar solvents such as aniline, nitrobenzene, phenol, phthalic anhydride, DMSO, and DMF upon heating. The reason for the low solubility and high melting point of indigo is bifurcated intra- and intermolecular hydrogen bonding [11], and face-to-face π–π stacking of parallel aromatic rings (Figure 2) [12].

**Figure 3:**
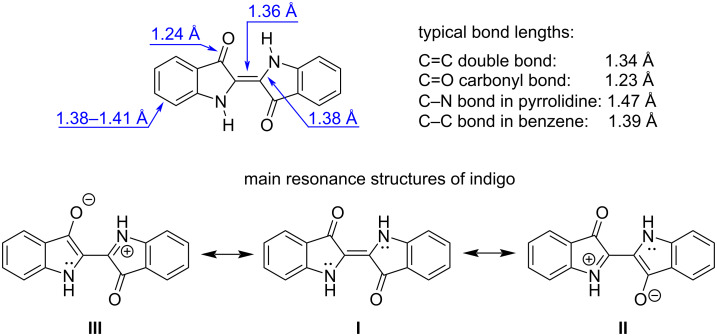
Bond length in the indigo molecule obtained from the single crystal X-ray analysis [12], the typical bond lengths in organic compounds [15] and the main resonance structures of indigo.

Single crystal X-ray diffraction analysis showed that the indigo molecule is almost planar and exists in the *E*-conformation. The central C=C bond and C=O bonds of the indoxyl groups are somewhat longer than typical double bonds of these types (Figure 3). On the contrary, the C–N bonds in indigo are much shorter than the single C–N bond in pyrrolidine, while the lengths of the C–C bonds in the fused benzene rings are approximately the same as in benzene (Figure 3). This indicates that in addition to structure **I**, which is traditionally used to depict indigo, intraionic resonance structures **II** and **III** with single C–O bonds and double C=N bonds make a pronounced contribution in the ground state of indigo (Figure 3) [13–14]. In the excited state, the resonance structures **II** and **III** with separated charges become predominant, while both benzene rings remain fully aromatic.

**Figure 4:**
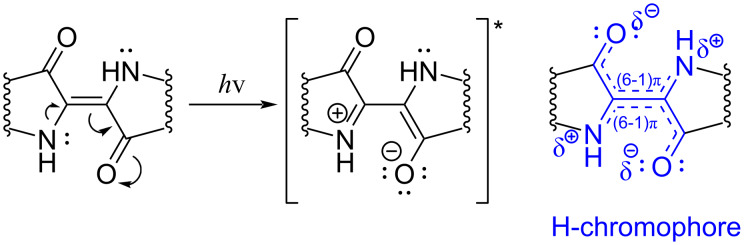
The structure of the indigo chromophore (H-chromophore, highlighted in blue), asterisk indicates the molecule in the excited state.

The indigo dye contains two heterocyclic indole ring systems, which are connected through a double bond and have 22 π-electrons [16–17]. However, only 10 π-electrons are involved in the main indigo chromophore comprising a C=C double bond substituted by two acceptors and two donor groups (Figure 4) [18]. Notably, the benzene rings make just a small contribution to the chromophore. In other words, the primary chromophore of indigo consists of two intersecting merocyanine structures connected by an ethylene bridge, and the electronic transition responsible for the color takes place within this structural element of the indigo molecule (Figure 4).

To emphasize the special nature of the indigo-type systems, Klessinger and Lüttke proposed to call their chromophores “H-chromophores” after the shape of the main structural fragment resembling the Latin letter “H” (Figure 4) [19]. The small HOMO–LUMO gap and, thus, the long-wavelength absorption are responsible for the purple color of indigo in vapors, which changes into deep blue color in the crystalline state due to formation of H-bonded associates (Figure 2) [20–22].

**Figure 5:**
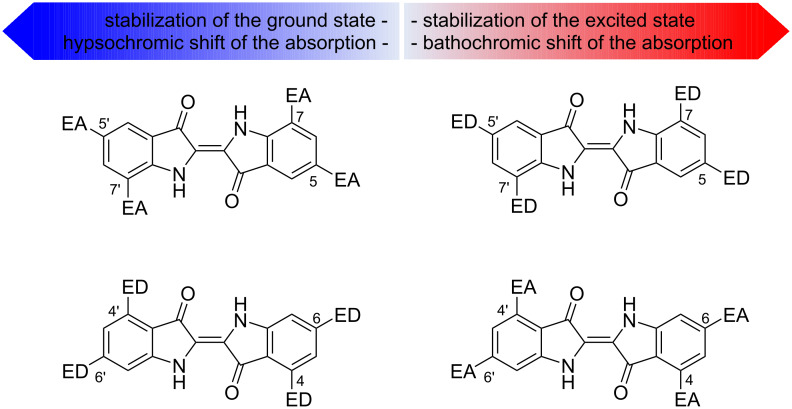
Influence of substituents in the benzene rings on the color of indigo derivatives.

As discussed above, the benzene rings play only a minor role in the formation of the color of indigo and its derivatives. However, electronic effects and especially the position of the substituents in the benzene rings have pronounced influence on the color of these dyes, as shown by the absorption maxima positions of the substituted indigo derivatives (Table 1) [23–24]. The introduction of the ED (electron-donating) substituents into the *para* (5,5')- or *ortho* (7,7')-positions to the electron-donating NH group results in a bathochromic shift of the absorption band and increases its intensity, while the presence of the EA (electron-accepting) substituents in these positions leads to a hypsochromic shift (Figure 5). On the contrary, the introduction of the ED substituents at the *para* (6,6')- or *ortho* (4,4')-positions to the electron-withdrawing C=O group causes a hypsochromic shift of the absorption band, while the presence of the EA substituents at these positions leads to a bathochromic shift (Figure 5). In addition, electron-donating substituents on the nitrogen atom produce bathochromic shifts, while electron-withdrawing groups at this position give hypsochromic shifts of the absorption maxima [24]. These effects can be easily explained considering the direction of the charge transfer in the H-chromophore of indigo upon excitation. Thus, in the excited state, the electron density shifts from the N towards the O atom (Figure 4). Therefore, an increase in the electron density on the N atom in the ground state due to the electronic effects of substituents facilitates charge transfer during excitation, which leads to a decrease in the energy of the transition and a bathochromic shift of the absorption band, and vice versa.

**Table 1:** Absorption maxima of indigo and its derivatives in C_2_Cl_4_ at 20 °C [23–24].

Substituents	λ_max_ / nm			

	4,4′-	5,5′-	6,6′-	7,7′-

none	605			
F	–	615	590	560
Cl	610	620	590	600
Br	610	621	585	605
I	620	610	590	605
CF_3_	605	–	–	580
NO_2_	–	580	635	–
Me	–	620	595	–
OMe	–	644	577	–

### Photochemistry of indigo

Despite thousands of years of use, the demand for indigo derivatives in the industry is still growing. The main reason for this is the unusual photostability and color fastness of indigo as industrial dye. This exceptional photostability of indigo and its derivatives has attracted significant scientific interest and, therefore, has been investigated comprehensively. To understand the photochemical and photophysical properties of indigo, a great number of theoretical and experimental studies [25–35] have been performed so far, which allowed to characterize excited state lifetimes, radiative and non-radiative relaxation pathways of the indigo chromophore [26–27,30–35] as well as to estimate ionization potentials and electronic structures [28–29], singlet oxygen generation capacity [31], QSAR properties [25], and others.

**Figure 6:**
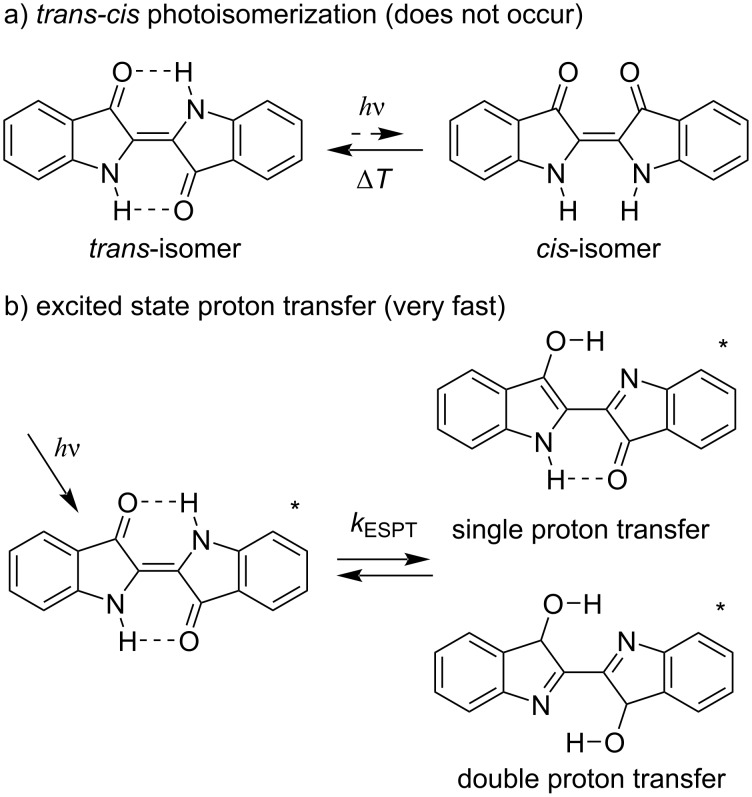
a) *E*–*Z* photoisomerization of indigo and b) photoinduced proton transfer in the excited state, asterisk indicates the molecules in the excited state.

In both solution and solid state, indigo exists in a planar *E*-form, which is more stable than the overcrowded and non-planar *Z*-form (Figure 6a) [23]. Irradiation of *E*-indigo, however, does not result in the photoisomerization into the *Z*-form due to the rapid proton transfer occurring in the excited state (ESPT) from the N atom towards the O atom (Figure 6b). Moreover, the data of the first systematic computational ab initio study of the molecular mechanism of the photostability of indigo [36] support these findings and additionally point out that the single proton transfer (SPT) is more favorable than the double proton transfer (DPT). Taking together, the main indigo scaffold is not photochromic. However, substitution of the hydrogen atom in the NH groups of indigo can change the photochemical behavior significantly.

### Photochromic indigo derivatives

**Figure 7:**
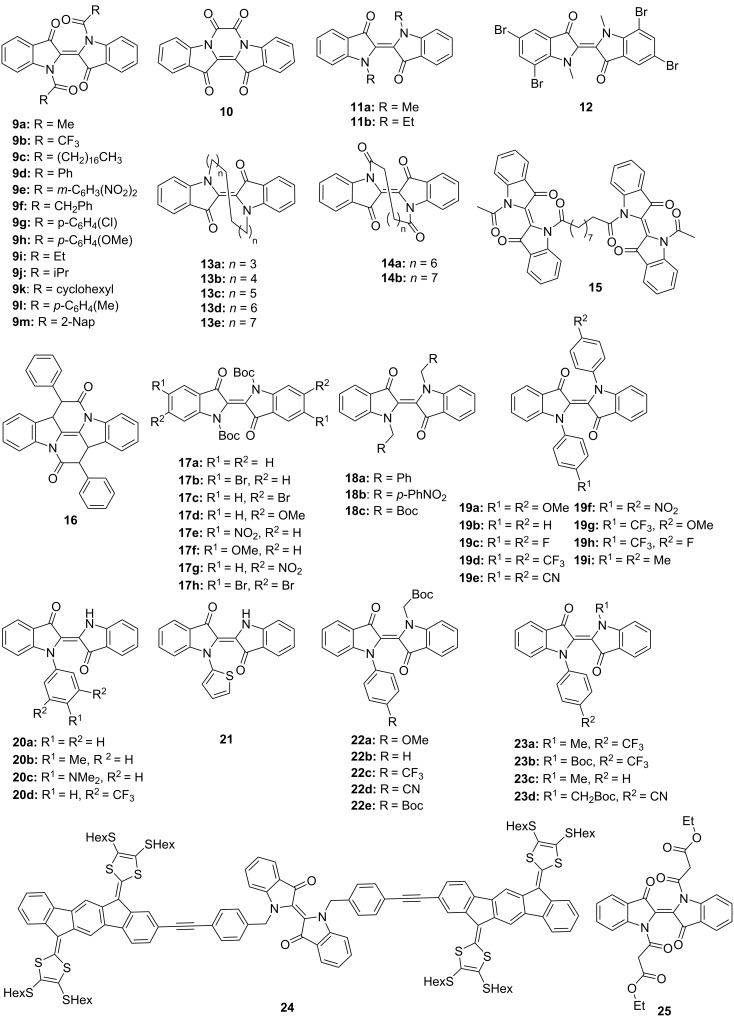
Structures of indigo derivatives discussed in this review.

**Figure 8:**
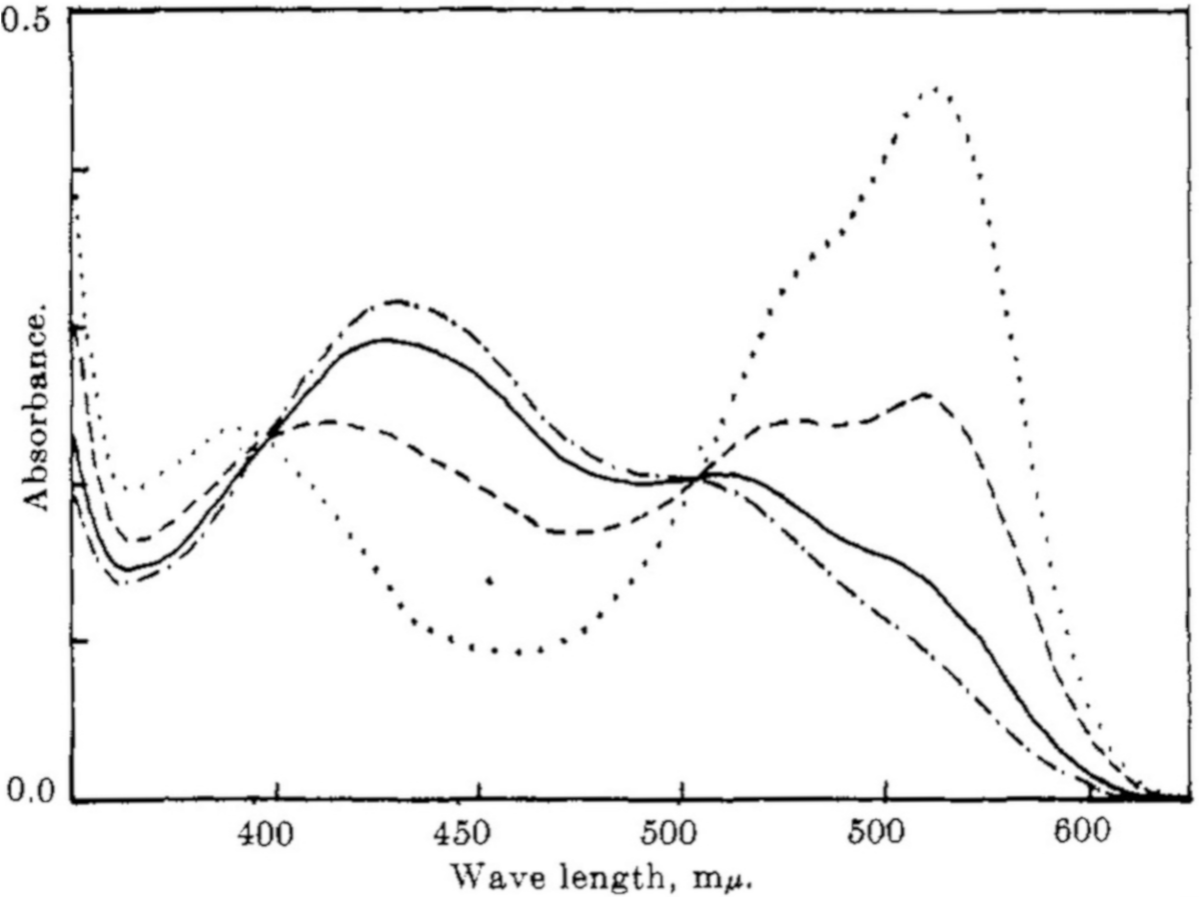
Photoswitching of *N,N'*-diacetylindigo (**9a**) in CCl_4_ (*c* = 17.1 µM; cell length = 5.0 cm) irradiated with blue light (λ_irr_ = 350–510 nm): dotted line; irradiated with white light: dashed line; irradiated with yellow light (λ_irr_ > 495 nm): solid line; irradiated with orange light (λ_irr_ > 520 nm): dash-dotted line. In all cases irradiation time *t* = 5 min. Reprinted with permission from ref. [38]. Copyright (1965) American Chemical Society.

In 1954, the first photochromic indigo derivative, namely *N,N'*-diacetylindigo (**9a**, Figure 7) was reported by Wyman and co-workers [37]. Interestingly, compound **9a** showed pronounced negative photochromism in benzene with the best *E*–*Z* conversion upon irradiation with yellow light (λ_irr_ > 520 nm), while in chloroform no photochromism was detected. The formation of the *Z*-isomer of **9a** in benzene was unambiguously proven by comparison with the oxalyl derivative **10** comprising the *Z*-double bond. Thus, the absorption band of *Z*-**9a** at about 430 nm closely matched the absorption maximum of the oxalyl indigo **10** (436 nm) pointing out the similarity of the chromophoric structures of both dyes [37]. Later, Wyman and Zenhäusern observed pronounced photochromic behavior of *N,N'*-diacetylindigo **9a** in CCl_4_ (Figure 8) [38].

#### Photochromic *N,N'*-dialkylindigos

In 1956, Wyman and Weinstein conducted elaborative spectroscopic studies on *N,N'*-dimethylindigo dye **11a** and found that compound **11a** underwent *E–Z* photoisomerization in benzene upon irradiation with yellow light and returned to *E*-isomer in darkness at room temperature within about 30 seconds [39]. One year later, Pummerer and Marondel attempted to reproduce this experiment and to detect the *Z*-isomer of *N,N'*-dimethylindigo **11a** upon irradiation with orange light in several solvents [40]. However, due to the technical limitations of the spectrometer, Pummerer and Marondel were not able to detect the short-living *Z*-**11a** in aromatic solvents such as benzene, bromobenzene and pyridine. Nevertheless, the *E–Z* isomerization of **11a** was observed in chloroform and carbon tetrachloride upon exposure to orange light. The lifetime of *Z*-**11a** in CCl_4_ was significant and the thermal backward reaction occurred in darkness with the restoration of the initial absorption of the *E*-isomer for 57% after 5 h and for 100% in 18 h. *N,N'*-Diethylindigo (**11b**) and *N,N'*-dimethyl-5,5',7,7'-tetrabromoindigo (**12**) showed similar photochemical isomerization in the chloroalkane solvents. Additionally, a slow photoreaction in benzene was detected for compound **12** [40]. 11 years later, Margerum and co-workers investigated the photochromic behavior of **11a** and **12** using the flash photolysis by a ruby laser (λ = 694 nm) in combination with photostationary methods [41]. The data on the absorption maxima, decay times, and extinction coefficients for *Z*-**11a** and *Z*-**12** obtained in these measurements significantly differed from those reported by Pummerer and Marondel [40]. In particular, the lifetime of *Z*-**11a** in CCl_4_ was found to be only 3.1 s versus 18 h measured previously. Nevertheless, it was obvious that *N,N'*-dialkylindigos were prone to the reversible photochemical isomerization. In addition, it was also found that the thermal backward *Z–E* conversion of **11a** and **12** accelerated in the presence of acid.

In 2017 and 2022, Hecht, Jacquemin and co-workers successfully synthesized a range of *N,N'*-disubstituted indigos, including *N,N'*-dialkyl derivatives, using novel approaches and investigated their photophysical properties, in particular, the thermal half-lives and the ratio of *Z*-isomers in the photostationary states [42–43]. The introduction of methyl and benzyl substituents in **11a** and **18a**, respectively, led to a bathochromic shift of the absorption maxima, while introduction of the *tert*-butyloxycarbonylmethyl substituent in **18c** yielded a slight hypsochromic shift due to the presence of the electron-withdrawing ester groups. Importantly, only the *Z*-form of **18c** was found to be stable enough with a half-life of 2.8 min at room temperature in acetonitrile (77% of *Z*-isomer in PSS) and 4.1 minutes in toluene (91% of *Z*-isomer in PSS) after irradiation with red light (λ_irr_ = 660 nm). The excited state dynamics of **18c** was later studied in detail by Nagasawa and co-workers using femtosecond time-resolved transient absorption spectroscopy [44].

**Figure 9:**
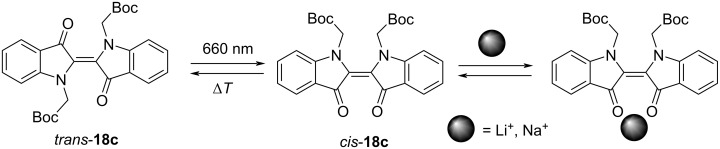
Photoisomerization of compound **18c** upon irradiation with red light and schematic representation of the complexation of the *Z*-isomer with metal cations.

In 2022, Qiao and co-workers attempted to extend the thermal relaxation half-life of compound **18c** by addition of cations such as Li^+^, Na^+^ and tetrabutylammonium (TBA^+^), which could be coordinated by two carbonyl groups of the indoxyl fragments in the *Z*-form (Figure 9) [45]. The longest half-life of 21.4 min for *Z*-**18c** along with the increase of the *E–Z* conversion in PSS from 32% to 75% was achieved in the presence of Li^+^ cations (500 equiv). At the same time, addition of TBA^+^ cations showed just a minimal impact on the stability of the *Z*-form comparable with the effect of counter anions and solvent polarity.

In 2021, Tsubaki and co-workers synthesized bridged *N*,*N′*-dialkyl-substituted indigos **13** and studied their thermal isomerization and photochemical (only for **13a**) isomerization [46]. Compounds **13** showed intrinsic planar chirality and their enantiomers could be separated by HPLC. Notably, upon thermal and photochemical isomerization of these compounds, no *Z*-isomers were detected and only the racemization took place. Such behavior was explained by the large energy gap between the ground states of the *E*- and *Z*-forms of indigo as well as low activation energy of inversion for derivatives **13**.

In the same year, Nielsen, Hecht and co-workers achieved a remarkable stabilization of the *Z*-isomer of *N,N'*-disubstituted indigo **24** by incorporating redox-active tetrathiafulvalene (TTF) residues (Figure 7), known for their ability to form intramolecular π-dimers upon two-electron oxidation [47]. Thus, upon irradiation of the oxidized molecule **24** with 660 nm light, the thermal half-life of the *Z*-isomer exhibited a significant increase, extending from 0.48 seconds to 22 hours at room temperature due to formation of the long-living intramolecular dimeric species.

#### Photochromic *N,N'*-diacylindigos

**Figure 10:**
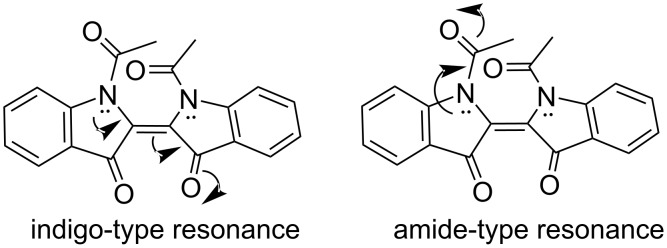
Schematic representation of indigo-type (left) and amide-type (right) resonances in *N,N'*-acetylindigo (**9a**).

After the initial report on the photochromic behavior of *N,N'*-diacetylindigo (**9a**) in 1954 [37], Wyman and Zenhäusern continued spectroscopic studies of the photoinduced *Z*-isomers of **9a** and *N*,*N*′-bis(trifluoroacetyl)indigo (**9b**) [38]. It was found that both derivatives **9a**,**b** formed unstable *Z*-isomers when exposed to light of different wavelengths in CCl_4_ (Figure 8). Because of the disrupted coplanarity of these *Z*-isomers, a pronounced hypsochromic shift was observed, which can be attributed to a weakening of the indigo-type resonance, while the amide resonance remained relatively unaffected (Figure 10).

One of the crucial problems accompanying the investigations of photoswitchable molecules are the situations when the photoisomerization product cannot be isolated in its pure state due to technical limitations or short lifetimes. In these cases, the experimental absorption spectrum of the photoproduct remains unknown. In 1967, Ernst Fischer proposed a mathematical method allowing to calculate the absorption spectrum of the photoinduced form B in the reaction of A ↔ B using experimental absorption spectra of the initial form A and two photostationary states (PSS) obtained at two different wavelengths [48]. In 1968, Ross and Blanc for the first time applied the Fischer method to calculate the extinction coefficients and quantum yields of the forward and backward isomerization of *N,N'*-diacetylindigo (**9a**) in toluene without isolation of the *Z*-form [49]. The Fischer method remains relevant up to date and is frequently used for the characterization of the photoinduced forms of various indigoid photoswitches [50–54].

**Figure 11:**
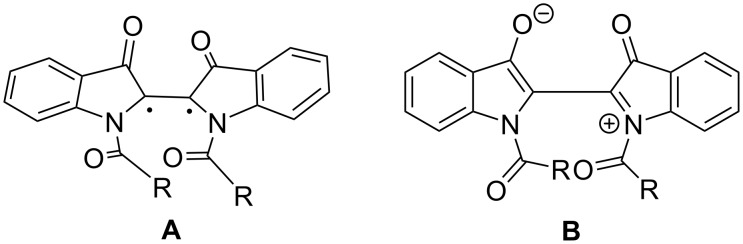
Suggested intermediates for the double bond cleavage for the thermal relaxation of *N,N'-*diacylindigos: (A) a biradical transient species, (B) a dipolar transient species.

To assess an effect of the size and structure of *N,N'*-diacyl substituents on the backward *Z–E* relaxation, in 1979, Omote and co-workers synthesized a series of *N,N*′-diacylindigo derivatives and isolated the *Z*-isomers of diacetyl- (**9a**), distearoyl- (**9c**), dibenzoyl- (**9d**), and bis(3,5-dinitrobenzoyl)indigo (**9e**) in the crystalline form [55]. It was found that the aliphatic substituents (diacetyl- and distearoyl-) supported faster relaxation rates than the aryl-containing substituents (dibenzoyl- and bis(3,5-dinitrobenzoyl)-). Moreover, addition of catalytic amounts of amines accelerated the backward *Z–E* isomerization of *Z*-**9a** and *Z*-**9d** in benzene solution. Interestingly, the order of acceleration was primarily dependent on the basicity of the catalyst, unless the steric hindrance of the amine became a significant factor, as is the case of diisopropylamine and trimethylamine. Compounds **9d** and **9e** are the first examples of photochromic *N,N'*-diacylindigos comprising aromatic substituents. In 1982, Kitao and co-workers synthesized a series of *N,N'-*diacylindigo derivatives **9a**, **9f**, **9i**, **9j**, **9m** and **25** and investigated their thermal *Z–E* isomerization in benzene and acetonitrile [56]. In this study, the values for the heat of isomerization of *N,N'*-diacylindigos were measured for the first time. Based on the previous reports, it was expected that inserting further methylene groups as a bridge between the two carbonyl carbons of the oxalyl group in compound **10** would alleviate the conformational restriction and fix the molecules in the *Z*-form. To prove this idea, in 1984, Omote and co-workers obtained intramolecularly bridged *N,N'*-azelaoylindigo (**14a**), *N,N'*-sebacoylindigo (**14b**), and intermolecularly bridged *N',N''*-sebacoylbis(*N*-acetylindigo) (**15**) and studied their *Z–E* thermal isomerization in comparison with *N,N'*-diacetylindigo (**9a**) [57]. The bridged compounds **14** and **15** showed *E–Z* photoisomerization upon irradiation by 550 nm light at room temperature and then underwent thermal *Z–E* relaxation in xylene at 81.8 °C. The azelaoyl substituent in the intermolecularly bridged *N,N'*-diacylindigo **15** did not have significant influence on the thermal *Z–E* isomerization compared to the acetyl group, while the sebacoyl substituent in **14b** extremely accelerated it. In contrast, the sebacoyl group in the intermolecularly bridged derivative **15** did not affect the thermal *Z–E* isomerization in comparison with *N,N'*-diacetylindigo (**9a**) due to the lack of steric restriction [57]. The thermal *Z–E* isomerization of *N,N'*-diacylindigos was further investigated by Sueishi and co-workers in 1985 [58]. In this study, the kinetic experiments for a range of derivatives **9a**, **9c**, **9d**, **9g**, **9i–m** bearing various *N,N'*-diacyl substituents in different solvents and under high pressures were performed. It was found that *N,N'*-dibenzoylindigos underwent the thermal relaxation much more slowly than the compounds without the aromatic ring in the acyl group. The biradical mechanism (Figure 11) was found to be the preferable pathway for the thermal *Z–E* isomerization of *N,N'*-diacylindigos.

In 1986, Kossanyi and co-workers investigated the relaxation processes of excited *E*-isomer of *N,N'*-diacylindigo dyes **9a**, **9d, 9g**, **9h** in comparison with the rigid molecules of oxalylindigo **10** (fixed in the *Z*-form) and cibalackrot (**16**) (fixed in the *E*-form) by nanosecond flash photolysis in combination with steady-state measurements [59]. It was found that the *E–Z* photoisomerization of **9a**, **9d, 9g**, **9h** occurred through a singlet mechanism upon direct excitation. However, the triplet state could be achieved by a sensitized reaction. At room temperature, a transient species that could be assigned to a triplet state was also observed for rigid molecules **10** and **16**, based on the quenching experiments and the fact that the same transient species could be found under sensitized conditions. Further insight in the excited state properties of derivatives **9a**, **9d, 9g**, **9h** and *N,N'*-dinitrobenzoylindigo **18b** were provided by de Melo and co-workers in 2020 using modern femtosecond spectroscopic techniques as well as TD-DFT calculations [60].

**Figure 12:**
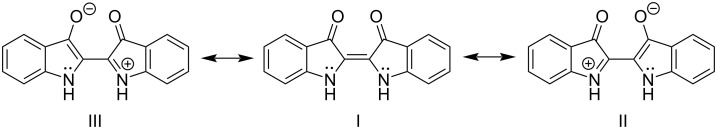
Zwitterionic resonance structures of *Z*-indigo.

In 1989, Takahashi and co-workers reported an important study of geometry and conformational stability of indigo and its *N,N'*-disubstituted derivatives [61–62]. Thus, unsubstituted indigo and oxalylindigo **10** displayed nearly planar geometry. At the same time, *N,N'*-diacetylindigo (**9a**)**, ***N,N'*-dimetylindigo (**11a**), and *N,N'*-azelaoylindigo (**14a**) exhibited significant twists around the central C=C double bond in both *E-* and *Z*-configurations. For example, the twisting angles for *N,N'*-dimethylindigo in the *E*- and *Z*-forms were 30.7° and 27.4°, respectively. Importantly, the twisting angle of the *E*-isomer is not always smaller than the one of the *Z*-isomer. Interestingly, no correlation between the positions of the absorption maxima of both *E-* and *Z*-isomers of the dyes **9a, 11a**, **14a** and the twisting angles or the C=C stretching frequencies was found. Therefore, the hypsochromic shift observed during the *E–Z* isomerization of *N,N'*-disubstituted indigos cannot be explained by a larger twisting angle in the *Z*-isomer [61]. As was mentioned above, the contribution of the zwitterionic resonance structures (Figure 3) increases in the excited state of indigo. This partial redistribution of charges, in turn, increases the electrostatic repulsion between the carbonyl groups in the *Z*-form (Figure 12) resulting in the larger energy gap between HOMO and LUMO and reduced stability for the *Z*-isomers in comparison with the *E*-forms [62].

In 2018, Nagasawa and co-workers provided a detailed investigation of the influence of solvent polarity and intermolecular hydrogen bonding on the photoisomerization of *N,N'*-diacetylindigo (**9a**) by transient absorption spectroscopy [63]. In principle, it was already known that the intermolecular hydrogen bonding with solvent molecules may hinder the photoisomerization of indigo derivatives [46]**.** Nagasawa and co-workers found that the photoisomerization of **9a** was inhibited by polar DMF due to formation of weak hydrogen bonds with formyl groups. However, even in low-polar ethyl acetate, *Z*-**9a** was not detected suggesting that the *E–Z* photoisomerization was slow and low efficient and could be easily hindered by rapid nonradiative de-excitation due to weak hydrogen bonding with the solvent. Notably, DMF was more effective than methanol in blocking the isomerization of **9a**.

**Figure 13:**
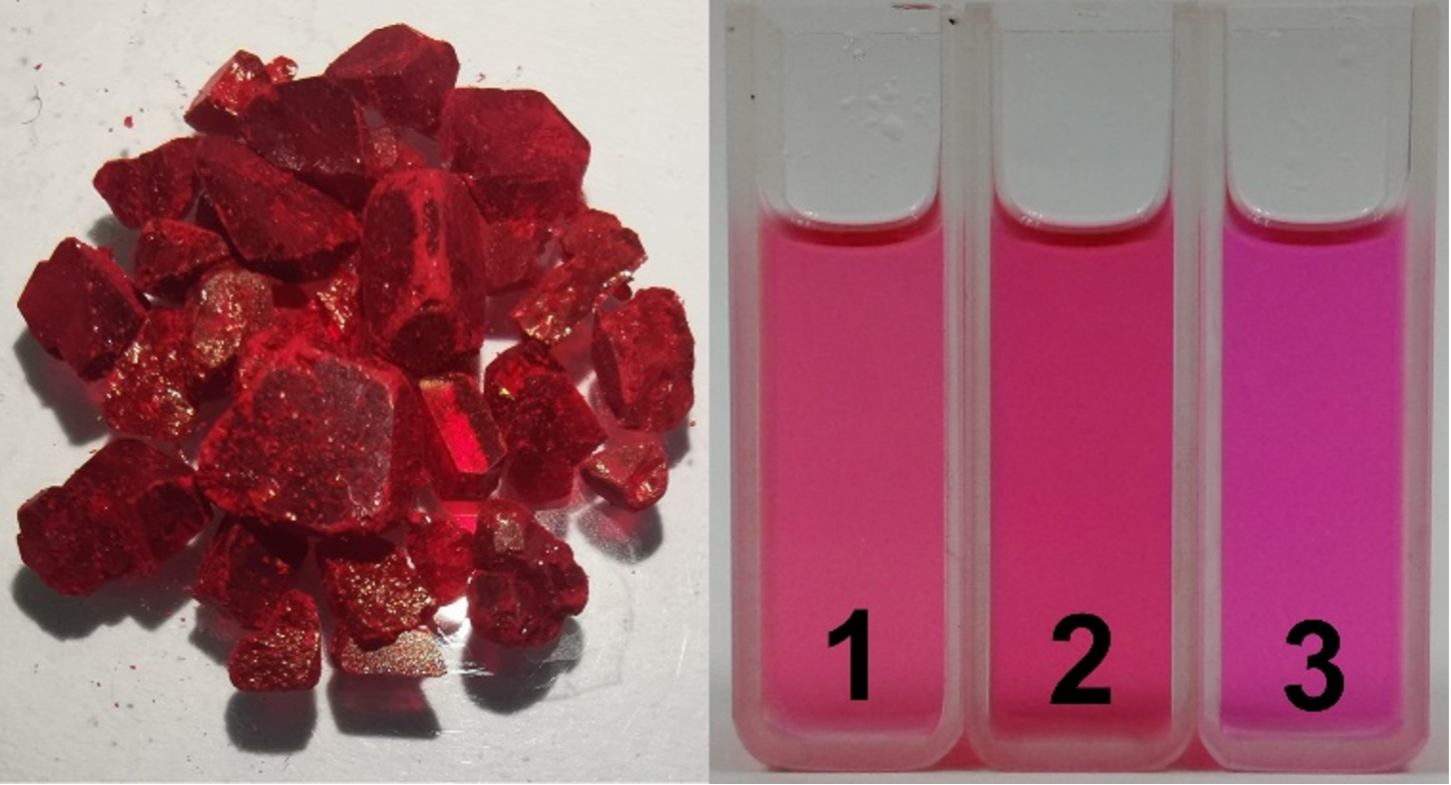
Photos of crystalline *N,N'*-di(Boc)indigo **17a** its solutions in 1) DMSO, 2) DMF, 3) *N*-methyl-2-pyrrolidone.

In 2015, Głowacki and co-workers successfully synthesized *N,N'*-di(*tert*-butoxycarbonyl)indigos **17a–c** for the first time [64]. The incorporation of bulky *tert*-butoxycarbonyl (Boc) substituents induced a notable strain on the central ethylenic carbon that in combination with the acceptor effect of the Boc groups resulted in the hypsochromic shift of the absorption of **17a** relative to the unsubstituted indigo yielding a magenta colored species (Figure 13). Additionally, the mentioned sterical strain substantially reduced the energy barrier for isomerization. Upon exposure to a 532 nm laser, compound **17a** exhibited pronounced negative photochromism. The thermal backward reaction occurred within 120 min in the dark in acetonitrile. The photoisomerization quantum yields for compounds **17a** (φ*_Z→E_* = 0.13, φ*_E→Z_* = 0.46), **17b** (φ*_Z→E_* = 0.01, φ*_E→Z_* = 0.02), and **17c** (φ*_Z→E_* = 0.06, φ*_E→Z_* = 0.20) were measured. The significant difference in the quantum yield values between the compounds was attributed to the increase in the population of triplet excited states because of spin–orbit coupling by bromine atoms. On the contrary with previous studies, it was found that the effect of proton and electron donors on the isomerization yields was negligible because of the Boc groups. A few years later, Koeppe and Schröder prepared several *N,N'*-di(Boc)indigo derivatives **17a–h** in order to study the effect of substituents on photoisomerization in solvents of different polarity [53]. It was found that, depending on the solvent, the thermal half-lives varied within a wide range from seconds to days. For example, for compound **17e**, the half-lives changed from 8 s (in MeOH) to 11 h (in toluene). In addition, the *Z*-form of compound **17d** was unusually stable in 1,4-dioxane, lasting for almost 6 days. The 2021, findings from the de Melo group confirmed that the photoisomerization of compound **17a** was a result of the absence of ESPT, in contrast to the mono-Boc substituted indigo derivative which exhibited ESPT [65].

#### Photochromic *N,N'*-diarylindigos

*N,N'*-Diarylindigos, a sub-type of *N,N'*-disubstituted indigos, recently attracted increased attention as red-light photoswitches. The investigation of the photophysical properties of these compounds began in 2017 with the work by Hecht, Jacquemin and co-workers [42]. In their study, a series of symmetrical as well as unsymmetrical *N,N'*-diaryl-substituted indigos **19a–h** was prepared and the photoisomerization behavior of these compounds was studied. Surprisingly, all *N,N'*-diarylindigos were obtained as mixtures of *E/Z* isomers. The computational study showed the negligible energy differences between the *Z*- and *E*-isomers of **19a–h** in the ground state, which explained the existence of the isomeric mixtures in the dark state. Interestingly, the initial *E*/*Z* ratio was restored when the compounds were left in darkness after irradiation with red light (660 nm). The time required for the restoration of the initial state varied from 58 s (in the presence of electron-donating group, **19a**) to 6.8 h (in the presence of electron-withdrawing group, **19f**) depending on the effect of the substituents in the aryl groups. Further insights into the photoisomerization mechanism of *N,N'*-diarylindigos were provided in 2022 using steady-state and time-resolved spectroscopy experiments along with theoretical calculations [43]. It was found that *N*-aryl substituents in combination with the polar solvents result in the rapid non-radiative deactivation upon excitation of the initial *E*-isomer of indigo. At the same time, *N*-aryl substituents significantly increased the barrier for the thermal backward isomerization of the *Z*-isomer yielding the long thermal half-lives of the photoinduced forms.

#### Photochromic mono-arylated indigos

In 2018, Dube and co-workers synthesized a range of mono-arylated indigo derivatives **20** and **21** and studied for the first time the photoisomerization of these compounds [66]. It was shown that despite the prerequisites for the occurrence of the excited-state proton transfer, monoarylated indigos underwent *E*–*Z* photoisomerization upon red-light irradiation at 625 nm. Remarkably, the thermal half-life of the *Z*-isomer of compound **20b** was sensitive to the presence of water. Thus, the thermal *Z*–*E* relaxation of **20b** could be significantly accelerated (ca. 300 times) by addition of water to organic solvent because of specific interactions between the NH groups and water molecules.

#### Photochromic *N*-aryl-*N'*-alkylindigos

In 2017 Hecht, Jacquemin, and co-workers prepared unsymmetrical indigo derivatives **22** bearing a –CH_2_Boc as *N'*-alkyl group and various *N*-aryl groups [42]. It was found that the electronic effect of the *para*-substituents in the *N*-aryl pendants had a crucial influence on the thermal half-life of the photoinduced *Z*-isomers. Thus, the electron-withdrawing groups (compounds **22c** and **22d**) not only prolonged the thermal half-life but also increased the *Z*-isomer content in the PSS compared with derivative **22b** with no substituents in the *N*-aryl ring. The presence of an electron-donating group in the *para*-position of the *N*-aryl group (compound **22a**), oppositely, resulted in the decrease of both the thermal half-life and the content of the *Z*-isomer in the PSS. Additionally, the effect of the *N'*-alkyl substituents in the presence of *para*-CF_3_ substituted *N*-aryl group was studied for compounds **22c**, **23a** and **23b**. In this case, the thermal half-lives and the content of the *Z*-isomer in PSS increased with the increase of the electron-withdrawing character of the *N'*-substituent (CH_3_ < CH_2_Boc < Boc) (Table 2) [42]. Further studies on the photoisomerization mechanism of *N*-aryl-*N'*-alkylindigos were provided in a follow-up publication in 2022 [43]. It was shown that the combination the *N*-aryl and *N'*-alkyl substituents in a single indigo molecule provided the fine tuning of the thermal half-lives of the photoinduced isomers without disruption of their red-shifted absorption.

**Table 2:** Photophysical and photochemical characteristics of *N*-aryl- *N'*-alkylindigos [42,66].

Compounds	**22a**	**22b**	**22c**	**22d**	**22e**	**23a**	**23b**

*λ*_max, _*_E_* (nm)	633	629	622	623	623	635	584
*λ*_max, _*_Z_* (nm)	593	578	572	567	577	595	525
*Z*-content in PSS (%)	11	62	56	71	75	38	80
*t*_(eq)1/2_	59 s	1.9 min	3.5 min	5.8 min	3.8 min	57 s	3.2 h

In 2022, Qiao and co-workers investigated the photochemical behavior of the unsymmetrically substituted *N*-aryl-*N'*-alkylindigo photoswitches **22b**, **23c** and **23d** upon addition of Li^+^ cations. These studies revealed that the presence of the electron-withdrawing groups on the aryl moiety decreased the charge density on the carbonyl groups and weakened the interaction of the *Z*-isomers with Li^+^ that accounted for the considerably longer thermal half-lives of *Z*-**22b** and *Z*-**23c** in comparison to *Z*-**23d** [45].

### Photochromism of the structural isomers of indigo

**Figure 14:**
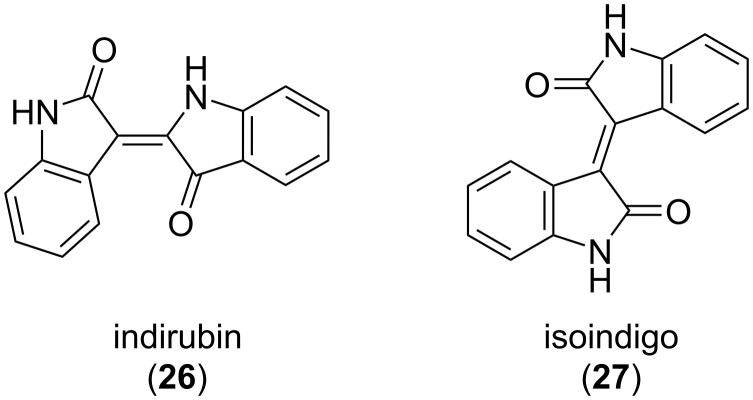
Structural isomers of indigo.

The indigo dye has two naturally occurring structural isomers, indirubin (**26**) and isoindigo (**27**), which differ in the attachment pattern of the two indole rings (Figure 14).

**Figure 15:**
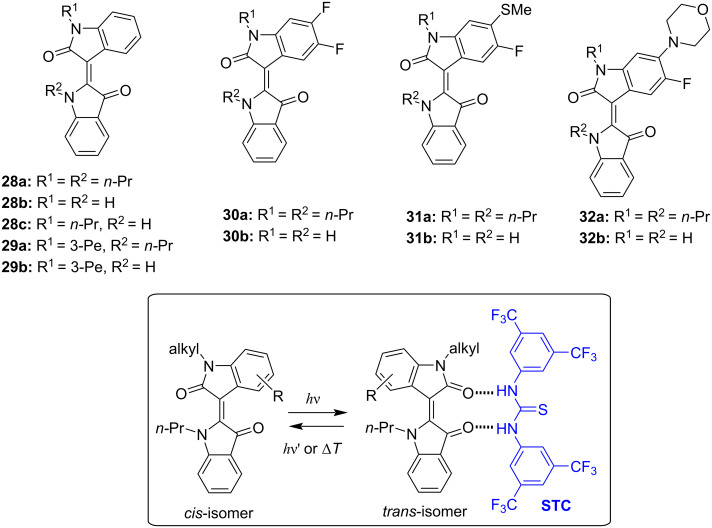
Photochromism of indirubin derivatives and supramolecular complexation of the *E*-isomers with Schreiner’s thiourea organocatalyst (STC).

Indirubin (**26**) is a purple colored dye that can be found in *Isatis tinctoria* and *Indigofera tinctoria* plants along with indigo and its derivatives or can be obtained as a metabolism product of some bacteria [68]. Given to the wide range of biological activities, including anticancer and anti-inflammatory effects, indirubin has been used as an active ingredient in the traditional Chinese medicinal recipe *Danggui Longhui Wan* [69–70]. The first synthesis of indirubin as a side product of attempts to synthesize indigo was described by Baeyer and Emmerling in 1870 [71]. The first report on the photochromism of indirubin derivatives **28**–**32** was provided by Dube and co-workers in 2021 (Figure 15) [72]. As expected, unsubstituted indirubin was not photochromic because of the fast photoinduced proton transfer taking place upon photoexcitation, while *N,N'*-dialkylated derivatives bearing additional substituents in the isatin ring showed pronounced negative photochromism upon irradiation with red light (625−650 nm). Supramolecular complexation with Schreiner’s thiourea organocatalyst (STC) allowed to reach better conversion with the isomeric ratio in PSS increased from 46% to 84%. The backward switching of the indirubin photoswitches took place upon irradiation of deep red light (730 nm).

**Figure 16:**
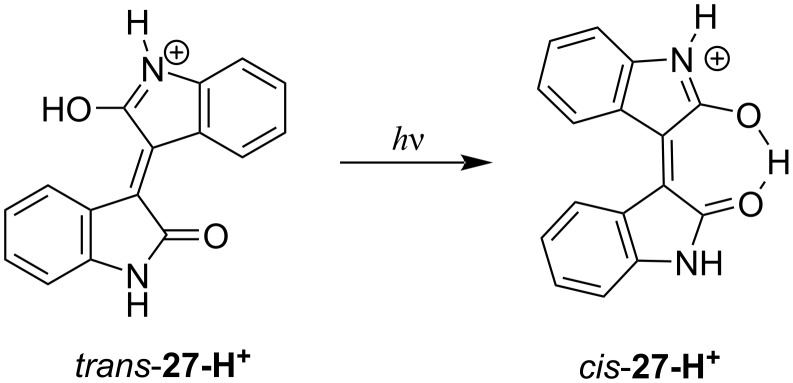
Photoisomerization of the protonated isoindigo.

Another structural isomer of indigo, the brown-colored isoindigo (**27**), was first synthesised by Laurent in 1842 [73]. Along with indigo and indirubin, isoindigo can be found in minor amounts in the leaves of *Isatis tinctoria* plant. Like indirubins, the isoindigo derivatives were initially studied as potential therapeutics with pronounced antitumor activity [74]. More recently, advantageous charateristics of isoindigos for the applications in organic photovoltaics (OPV) [75] and optoelectronics [76] were revealed. The isoindigo derivatives are photostable, and no photochromism was reported for these compounds till 2019, when Oomens and co-workers provided an evidence for the *E–Z* photoisomerization of the protonated isoindigo in the gas phase (Figure 16) [77]. The backward *Z–E* photoisomerization of the protonated isoindigo was not studied. To the best of our knowledge, no further reports on the photochromism of isoindigo and its derivatives were published up to now.

### Applications

Despite significant fundamental progress in the understanding of the structure–property relationship of photoswitchable indigo derivatives, the prospective applications of these compounds still remain underexplored. However, first attempts to commercialize indigoid photochromes were made 50 years ago. Thus, in 1970s–1980s, several patents related to the use of indigo photoswitches were issued. For instance, in 1977, a group of inventors working for Battelle Development Corporation, published a patent “Catalytic extraction of stored solar energy from photochemicals”, where the *Ν,Ν'*-diacylindigo derivatives were utilized as an energy storage element [78]. The mechanism of the energy storage relied upon the photoinduced *E–Z* isomerization of the indigo derivatives (energy accumulation) and the follow-up catalytic or heat-triggered *Z–E* reaction (energy release). Another example of a patent is “Sunlight-energy-storing substances and sunlight-energy-storing method using the same”, published in 1983 by Matsushita Electric Industrial Corporation [79], in which the inventors succeeded in the synthesis of water-soluble indigo derivatives and applied these compounds as solar energy storage systems using the aforementioned energy storage mechanism. However, detailed studies on kinetics and thermodynamics of the photoisomerization and thermal relaxation of *Ν,Ν'*-diacylindigo derivatives **9a**, **9d**, **9g** and **9i**, reported in 1984 by Pouliquen and co-workers, made the use of photochromic indigos as energy storage systems questionable [80].

Nowadays, indigo derivatives have gained increased attention as red-light photoswitches regarding their high application potential in biological systems and interactive "smart" materials that can adjust to environmental variations and respond to external light stimuli in a highly controllable manner. The prospective use of the indigoid photoswitches in photopharmacology [81–86] is especially appealing due to the possibility of performing the switching within the biooptical transparency window (650–900 nm) [87]. Additionally, significant structural differences (180° flip) between the *E*- and *Z*-isomers of the compounds provide a prerequisite of light-controllable geometry control in biomolecular assemblies as well as precise switching of the biological activity of the photoswitchable derivative. Along these lines, in 2023, Leung and co-workers prepared amphiphilic indigo-based photoswitches and incorporated them into vesicles [88]. Irradiation of the vesicles with red light resulted in the *E–Z* isomerization of the indigo derivatives that led to the disassembly of well-shaped vesicles and formation of irregular aggregates. The thermal *Z–E* relaxation of the indigo scaffold provided the restoration of the vesicle structure. The system contributed to the development of red-light-controllable biomedical soft materials. A possibility to apply photochromic mono-arylated indigo derivatives as water sensors with high sensitivity was demonstrated by Dube and co-workers in 2018 [66]. The detection principle relied on a remarkable acceleration (up to 300 times) of the thermal *Z–E* relaxation of compound **20b** in the presence of water in organic solvent. In 2023, Huang, Hecht, Priimagi and co-workers provided the first study of the photoswitching of *Ν,Ν'*-derivatized indigos in polymer films of different rigidity and described the molecular design strategy allowing to improve the photoswitching performance within the polymer matrix [67]. Based on the obtained results, the authors are aiming to translate the photoswitching effect to the macroscopic level and to achieve the modulation of macroscopic properties of materials by red light.

## Conclusion

From the first report on the photochromic *N,N'*-diacetylindigo in 1954 to the present time, the range of photochromic indigo derivatives has been significantly extended. In particular, the growing scientific interest, accompanied by a rapid increase in the number of publications on these molecules over the past six years, has brought this area of research to a new level of complexity and promise. The photochromic indigos represent a relatively rare type of visible-light-responsive T-type photoswitches demonstrating negative photochromism. The absorption of the photoswitchable indigos covers the spectral range from green to NIR light (≈550–700 nm) making these compounds especially attractive for the design of new biocompatible photochemical tools due to the possibility of performing the switching close to or within the biooptical transparency window (650–900 nm) [87]. The negative photochromism of indigos, which implies a blue-shift of absorption during the photoreaction, is advantageous for applications in bulk materials because the photoisomers have decreased absorbance at the excitation wavelength [9]. Depending on the substitution pattern, the quantum yields for the *E–Z* photoisomerization of indigo photochromes vary from 0.001 to 0.46 and the thermal half-lives of the photoisomers range from seconds to days. Detailed photophysical and photochemical studies have provided insight into the photoswitching mechanisms of indigo derivatives and enabled control of their photochemical properties through targeted design of the molecular structure. The toolkit of available synthetic methods makes several types of indigo photoswitches affordable and synthetically accessible. At the same time, the need to develop new synthetic approaches to obtain tailored indigo derivatives remains high [9]. In contrast to the recent review by Hecht and Huang [9] providing a detailed analysis of the synthetic methods and describing all types of the photochemical reactions of indigo derivatives (*E–Z* photoisomerization, photoinduced proton and electron transfer), our review is focused on the photoswitchable indigo derivatives undergoing the *E–Z* photoisomerization in view of historical development perspective of these compounds. Additionally, we highlight the recent advances in the photoswitching of the structural derivatives of indigo (indirubin and isoindigo) as well as outline the reported applications of photochromic indigos. Overall, we hope that this review will be useful in summarizing the advances and challenges as well as provide a platform for new scientific ideas and discoveries that will promote the applications of photochromic indigo derivatives in various fields from materials science and organic electronics to biology and medicine.

## Data Availability

Data sharing is not applicable as no new data was generated or analyzed in this study.
